# Severity of Carpal Tunnel Syndrome and Diagnostic Accuracy of Hand and Body Anthropometric Measures

**DOI:** 10.1371/journal.pone.0164715

**Published:** 2016-10-21

**Authors:** Mauro Mondelli, Andrea Farioli, Stefano Mattioli, Alessandro Aretini, Federica Ginanneschi, Giuseppe Greco, Stefania Curti

**Affiliations:** 1 EMG Service, Local Health Unit no.7, Siena, Italy; 2 Department of Medical and Surgical Sciences, University of Bologna, Bologna, Italy; 3 Department of Medical, Surgical and Neurosciences, University of Siena, Siena, Italy; 4 EMG Service, Local Health Unit no.7, “Nottola” Hospital, Montepulciano, Siena, Italy; Universita degli Studi di Napoli Federico II, ITALY

## Abstract

**Objective:**

To study the diagnostic properties of hand/wrist and body measures according to validated clinical and electrophysiological carpal tunnel syndrome (CTS) severity scales.

**Methods:**

We performed a prospective case-control study. For each case, two controls were enrolled. Two five-stage clinical and electrophysiological scales were used to evaluate CTS severity. Anthropometric measurements were collected and obesity indicators and hand/wrist ratios were calculated. Area under the receiver operating characteristic curves (AUC), sensitivity, specificity, and likelihood ratios were calculated separately by gender.

**Results:**

We consecutively enrolled 370 cases and 747 controls. The wrist-palm ratio, waist-hip-height ratio and waist-stature ratio showed the highest proportion of cases with abnormal values in the severe stages of CTS for clinical and electrophysiological severity scales in both genders. Accuracy tended to increase with CTS severity for females and males. In severe stage, most of the indexes presented moderate accuracy in both genders. Among subjects with severe CTS, the wrist-palm ratio presented the highest AUC for hand measures in the clinical and electrophysiological severity scales both in females (AUC 0.83 and 0.76, respectively) and males (AUC 0.91 and 0.82, respectively). Among subjects with severe CTS, the waist-stature ratio showed the highest AUC for body measures in the clinical and electrophysiological severity scales both in females (AUC 0.78 and 0.77, respectively) and males (AUC 0.84 and 0.76, respectively). The results of waist-hip-height ratio AUC were similar.

**Conclusions:**

Wrist-palm ratio, waist-hip-height ratio and waist-stature ratio could contribute to support the diagnostic hypothesis of severe CTS that however has to be confirmed by nerve conduction study.

## Introduction

Several epidemiological studies have investigated the association between anthropometric characteristics and the risk of carpal tunnel syndrome (CTS), the most common peripheral A mononeuropathy [1–5]. large body of literature supports a causal association between overweight or obesity and CTS [6–16]. Moreover, there is evidence that anatomical characteristics of the hand-wrist system may modulate the risk, and perhaps the severity, of CTS [10,11,13,17–24].

As anthropometric measures can be easily collected alongside symptoms and signs during clinical examinations, they could be proposed as a screening tool to detect subjects at risk of CTS. To explore this hypothesis, we previously tried to verify the existence of optimal cutoff values for anthropometric measurements to identify individuals with CTS. We analyzed several anthropometric indexes using receiver operating characteristic (ROC) curves and we found that all the studied variables were clearly associated with CTS [25]. However, none of the estimated areas under the ROC curves (AUC) was above 0.75 and it was not possible to identify cutoff values characterized by high sensitivity and specificity at the same time. Hence, due to the high proportion of false positive and false negative test results, we did not recommend the use of anthropometric characteristics as screening tools for CTS [25]. Nevertheless, our previous analysis was based solely on the presence or absence of CTS, ignoring the clinical or electrophysiological severity of the disease.

Now we hypothesize that the accuracy of selected anthropometric indexes to detect CTS may be higher in presence of severe disease. Hence, we present a reanalysis of our data aimed at studying the diagnostic properties of hand/wrist and body measures according to validated clinical and electrophysiological severity scales.

## Patients and Methods

### Study design and selection of cases and controls

We used the same methods and data described in two our previous studies [25,26]. The enrolment of cases and controls and the details of the electrophysiological methodology have been extensively reported elsewhere [25,26].

Consecutive patients admitted to three electromyography (EMG) laboratories reporting symptoms of the upper limbs were enrolled in the study. For the identification of incident cases of CTS, the diagnosis was based on clinical findings and delay of distal conduction velocity of the median nerve according to the consensus criteria for the classification of CTS in epidemiological studies [27]. The clinical diagnosis of CTS was performed following the recommendations of the American Academy of Neurology [28].

For the identification of controls, they were sampled among patients admitted to the same laboratories for complaints other than CTS. For each case, two controls were enrolled.

The following exclusion criteria were applied for the cases and controls: surgery of the upper limb; radiculopathy; polyneuropathy; amyotrophic lateral sclerosis; diabetes; rheumatic and thyroid diseases; renal failure; gout; history of alcoholism; presence of malignancy in the previous five years; hand/wrist trauma (with or without fracture); onset of CTS symptoms during pregnancy or lactation; and previous intake of medication considered toxic to the peripheral nervous system.

The ethics committee of Health Unit no.7 of Siena, Italy, approved the study and all patients gave written informed consent.

### Evaluation of clinical and electrophysiological severity of CTS

Clinical assessment of CTS severity was evaluated using a validated five-stage scale [29]. This scale was based on the timing of any type of paraesthesia complained in the previous two weeks, the presence of objective sensory deficits, the strength of opposition and abduction of the thumb and status of the thenar eminence muscles. In particular, the five stages of progressive clinical severity are: I, paraesthesia only at night and/or on waking in any part of the hand innervated by the median nerve; II, paraesthesia during the day even in case of transient diurnal symptoms after repetitive movements or prolonged postures; III, any degree of sensory deficit in any region of the hand supplied by the median nerve; IV, hypotrophy and/or motor weakness of the median-supplied thenar muscles; V, atrophy and/or plegia of the same muscles.

We evaluated the electrophysiological severity of CTS using a validated five-stage scale [30]. This scale evaluates the presence or absence of motor and sensory response, and normal or abnormal sensory conduction velocity (SCV), distal motor latency (DML) of the median nerve, and comparative nerve conduction velocity testing. In particular, the five stages of progressive electrophysiological severity scale are: I, normal DML and digit-wrist segment SCV (i.e. in the third digit-wrist and in the fourth digit-wrist (M4) tracts) and abnormal at least two of the following comparative tests: difference between the latencies of the median and ulnar nerves in 8 cm palm-to-wrist segment, difference between SCV of the median and ulnar nerves in the fourth digit-wrist tract; difference between SCV of the median and radial nerves in the first digit-wrist tract; difference between second lumbrical-interosseous muscles DML; difference between abductor pollicis brevis-abductor digiti minimi muscles DML; II, slowing of median digit-wrist segment SCV and normal DML; III, slowing of digit-wrist segment SCV and DML delay; IV, absence of sensory nerve action potential (SNAP) in digit-wrist segment (at least M4) and DML delay; V, absence of SNAP and compound muscle action potential.

### Hand and body anthropometric measurements

The collection of the anthropometric measurements was extensively described elsewhere [25,26]. For the purpose of the present analysis, we focused on selected hand and body measures.

With respect to hand/wrist measures, we evaluated the following ratios and indexes:

1) wrist ratio: wrist depth/wrist width [10,13,17,19,21,22]; 2) hand ratio: hand length/palm width [1,21]; 3) shape index: palm width x 100/hand length [11]; 4) digit index: third digit length x 100/hand length [11]; 5) wrist-palm ratio: wrist depth/length palm [19,23]; and 6) hand length-height: hand length/height [11].

Regarding body measures, we considered the following ratios and indexes: 1) body mass index (BMI): weight (kg)/height (m)^2^; 2) a body shape index: waist circumference/body mass index^2/3^ x height^1/2^ [31]; 3) waist-hip ratio: waist circumference/hip circumference [32]; 4) waist-stature ratio: waist circumference/height; and 5) waist-hip-height ratio: waist-stature ratio/height.

In case of patients who reported bilateral symptoms, we measured the hand with more severe symptoms. In the event of no difference between sides, we measured the dominant hand.

We tested the inter- and intraexaminer agreement of the selected anthropometric measures and reported the results elsewhere [26].

### Statistical analysis

Our database is reported in S1 Table, We aimed at investigating whether the diagnostic properties of selected anthropometrics indexes vary depending on clinical and electrophysiological severity stages of CTS. After preliminary analysis, the cases in stage I and II and those classified in stage IV and V of clinical and electrophysiological severity scales were grouped in two categories since the number of cases in the single stages was too small to obtain reliable statistical results. Therefore, CTS cases were classified into three groups of severity: mild (stages I/II); moderate (stage III); and severe (stages IV/V). We calculated the Spearman’s rank correlation coefficients to evaluate the strength of the association between the clinical and the electrophysiological severity scale.

The assumption of normality of the variables was tested through the Kolmogorov-Smirnov test with Lilliefors correction. Descriptive statistics of body and hand/wrist ratios were presented as mean and standard deviation (SD) according to clinical and electrophysiological severity of CTS. Data were analyzed separately for males and females. Trends across ordered groups were analyzed with the Cuzick non-parametric test (continuous variables) or with a score test for linear trend of the log odds (dichotomous variable).

To assess the diagnostic accuracy of the selected anthropometric indexes, we firstly used the optimal cut-off points published in our previous study performed on the same sample of subjects [25] to calculate the numbers of positive patients by CTS severity. Secondly, we calculated sensitivity, specificity, positive likelihood ratio (LR+) and negative likelihood ratio (LR-) within each stratum. Thirdly, we estimated the stratum-specific AUC, computed using the trapezoidal rule, and asymptotic normal confidence intervals. Then, we compared the AUC using the test for equality suggested by DeLong and colleagues [33]. Finally, we plotted the ROC curves for those two variables that showed the best accuracy properties in our previous study among hand/wrist and body measures (i.e. wrist-palm ratio and waist-stature ratio) [25] and we estimated the Bonferroni-adjusted p-values for pairwise comparisons across severity strata.

The AUC ranges from 0 to 1.0 with a value of 0.5 representing discrimination no better than chance. AUC values were interpreted using Swets suggestion’s [34]: 0.5 to 0.7, low accuracy; 0.7 to 0.9, moderate accuracy; >0.9, high accuracy. Likelihood ratios represent the change in the odds of a positive (LR+) or negative (LR–) diagnosis after testing, respectively, negatively or positively. LRs can be interpreted against the following reference values: LR+ >10 or LR–<0.1, large change in the probability of disease; LR+ between 5 and 10 or LR– 0.1 and 0.2, discrete change in the probability of disease; LR+ between 2 and 5 or LR–between 0.2 and 0.5, small but important change in the probability of disease; LR+ <2 or LR–>0.5, minimal practical utility.

All analyses were stratified by gender and performed using STATA 12.1 (College Station, TX, USA) software package. An alpha error of 0.05 was accepted.

## Results

Our study enrolled 370 cases and 747 controls. Mean age was 54.4 ± 15 years for female cases (n = 250) and 51.8 ± 16.6 years for female controls (n = 474). Among males, mean age was 57.9 ± 16.3 years for cases (n = 120) and 50.3 ± 16.3 years for controls (n = 273).

The Spearman’s rho coefficient between clinical and electrophysiological score was 0.59 for females and 0.65 for males, respectively.

Table 1 reported the main characteristics of the study population according to clinical and electrophysiological severity scales among females and males. A significant trend was present for almost all hand/wrist and body measures in both genders. In particular, the difference between the values observed among controls and cases was higher in the severe stage than in the moderate and mild stages for both clinical and electrophysiological score.

**Table 1:** Characteristics of the study population by gender and clinical/electrophysiological score.

	**FEMALES (N = 724)**															
			**Cases: clinical score**							**Cases: electrophysiological score**						
	**Controls***(N = 474)*		*Mild(N = 149)*		*Moderate (N = 72)*		*Severe (N = 29)*			*Mild (N = 84)*		*Moderate (N = 123)*		*Severe (N = 43)*		
**Variable**	*Mean*	*(SD)*	*Mean*	*(SD)*	*Mean*	*(SD)*	*Mean*	*(SD)*	*P trend*^a^	*Mean*	*(SD)*	*Mean*	*(SD)*	*Mean*	*(SD)*	*P trend*^a^
Age (years)	51.8	(16.6)	51.5	(14.5)	55.2	(12.8)	67.4	(15.5)	<0.001	49.8	(13.6)	54.0	(13.7)	64.7	(16.4)	<0.001
*Hand/wrist measure*																
Wrist ratio	0.704	(0.043)	0.708	(0.046)	0.719	(0.037)	0.737	(0.040)	<0.001	0.708	(0.044)	0.715	(0.043)	0.727	(0.042)	<0.001
Hand ratio	2.28	(0.12)	2.24	(0.13)	2.22	(0.13)	2.18	(0.12)	<0.001	2.23	(0.12)	2.23	(0.14)	2.20	(0.11)	<0.001
Shape index	44.0	(2.3)	44.9	(2.5)	45.1	(2.7)	46.1	(2.6)	<0.001	45.0	(2.6)	44.9	(2.7)	45.6	(2.3)	<0.001
Digit index	43.0	(1.2)	43.5	(1.3)	42.9	(1.7)	42.9	(1.7)	0.183	43.5	(1.4)	43.3	(1.5)	43.0	(1.6)	0.100
Wrist-palm ratio	0.379	(0.033)	0.387	(0.030)	0.397	(0.034)	0.418	(0.024)	<0.001	0.387	(0.030)	0.393	(0.034)	0.409	(0.026)	<0.001
Hand length/height	1.09	(0.05)	1.08	(0.05)	1.09	(0.05)	1.09	(0.06)	0.222	1.08	(0.05)	1.08	(0.05)	1.09	(0.05)	0.331
*Body measure*																
Body mass index	24.7	(4.5)	26.0	(4.3)	26.7	(4.8)	27.8	(4.5)	<0.001	25.8	(4.7)	26.3	(4.4)	27.8	(4.0)	<0.001
A Body shape index	0.790	(0.058)	0.800	(0.058)	0.803	(0.059)	0.826	(0.059)	0.001	0.795	(0.058)	0.804	(0.059)	0.821	(0.057)	<0.001
Waist-hip ratio	0.835	(0.071)	0.853	(0.076)	0.862	(0.066)	0.886	(0.055)	<0.001	0.848	(0.065)	0.858	(0.076)	0.886	(0.063)	<0.001
Waist-stature ratio	0.529	(0.077)	0.556	(0.078)	0.569	(0.073)	0.609	(0.076)	<0.001	0.549	(0.082)	0.564	(0.074)	0.602	(0.070)	<0.001
Waist-hip-height ratio	0.523	(0.052)	0.536	(0.053)	0.547	(0.048)	0.575	(0.048)	<0.001	0.533	(0.049)	0.542	(0.053)	0.570	(0.051)	<0.001
	**MALES (N = 393)**															
			**Cases: clinical score**							**Cases: electrophysiological score**						
	**Controls** *(N = 273)*		*Mild (N = 58)*		*Moderate (N = 38)*		*Severe (N = 24)*			*Mild (N = 24)*		*Moderate (N = 64)*		*Severe (N = 32)*		
**Variable**	*Mean*	*(SD)*	*Mean*	*(SD)*	*Mean*	*(SD)*	*Mean*	*(SD)*	*P trend*^a^	*Mean*	*(SD)*	*Mean*	*(SD)*	*Mean*	*(SD)*	*P trend*^a^
Age (years)	50.3	(16.3)	53.7	(15.4)	54.6	(14.9)	73.4	(10.7)	<0.001	47.7	(13.8)	56.2	(15.2)	69.1	(13.9)	<0.001
*Hand/wrist measure*																
Wrist ratio	0.699	(0.042)	0.704	(0.031)	0.727	(0.044)	0.719	(0.033)	<0.001	0.706	(0.035)	0.718	(0.038)	0.714	(0.038)	0.001
Hand ratio	2.24	(0.13)	2.14	(0.10)	2.12	(0.14)	2.05	(0.14)	<0.001	2.15	(0.11)	2.12	(0.12)	2.09	(0.15)	<0.001
Shape index	44.8	(2.6)	46.8	(2.3)	47.3	(3.0)	49.1	(3.5)	<0.001	46.7	(2.3)	47.4	(2.6)	48.2	(3.7)	<0.001
Digit index	42.9	(1.4)	43.0	(1.3)	43.2	(1.3)	43.1	(1.7)	0.091	43.5	(1.3)	43.0	(1.2)	42.9	(1.7)	0.202
Wrist-palm ratio	0.382	(0.028)	0.397	(0.027)	0.413	(0.029)	0.438	(0.040)	<0.001	0.398	(0.031)	0.409	(0.031)	0.422	(0.038)	<0.001
Hand length/height	1.10	(0.05)	1.09	(0.04)	1.09	(0.05)	1.09	(0.05)	0.037	1.08	(0.05)	1.09	(0.04)	1.09	(0.04)	0.039
*Body measure*																
Body mass index	26.3	(3.7)	27.6	(3.6)	28.2	(4.4)	29.9	(3.7)	<0.001	27.4	(3.5)	28.4	(4.4)	28.6	(3.4)	<0.001
A Body shape index	0.818	(0.058)	0.824	(0.044)	0.820	(0.043)	0.849	(0.051)	0.006	0.818	(0.042)	0.823	(0.047)	0.844	(0.043)	0.007
Waist-hip ratio	0.926	(0.082)	0.951	(0.060)	0.960	(0.061)	0.972	(0.061)	<0.001	0.952	(0.074)	0.958	(0.057)	0.962	(0.058)	<0.001
Waist-stature ratio	0.548	(0.065)	0.575	(0.056)	0.575	(0.710)	0.635	(0.065)	<0.001	0.566	(0.058)	0.584	(0.068)	0.610	(0.066)	<0.001
Waist-hip-height ratio	0.535	(0.052)	0.559	(0.044)	0.552	(0.039)	0.587	(0.042)	<0.001	0.538	(0.049)	0.548	(0.050)	0.572	(0.048)	<0.001

Classification of stages: mild, stage I-II; moderate, III; severe, stage IV-V.

^a^Cuzick non-parametric test for trend across ordered groups.

Similar results were obtained when optimal cut-offs of anthropometric factors were used (Table 2). These cut-offs aimed at discriminating patients with and without CTS were identified in a previous study [25]. With respect to hand/wrist measurements, the wrist-palm ratio showed the highest proportion of cases with abnormal values in the severe stage of CTS for both clinical and electrophysiological severity scale in both genders (i.e. 93% of positive cases for the clinical severity scale and 86% for the electrophysiological severity scale among females; 95% and 81% among males, respectively). Among body measurements, the waist-hip-height ratio and waist-stature ratio reported the highest proportion of cases above the cut-offs in the severe than in the moderate and mild stages for both clinical and electrophysiological severity scale in both genders. For instance, 86% of positive cases classified in the severe stage among females had abnormal waist-hip-height ratio in the clinical score and 84% in the electrophysiological score. Among males, the proportion of positive cases was 92% for the clinical score and 81% for the electrophysiological score, respectively.

**Table 2:** Number of subjects above the established cut-offs by gender and clinical/electrophysiological score.

	**FEMALES (N = 724)**																
				**Cases: clinical score**							**Cases: electrophysiological score**						
		**Controls** *(N = 474)*		*Mild (N = 149)*		*Moderate (N = 72)*		*Severe (N = 29)*			*Mild (N = 84)*		*Moderate (N = 123)*		*Severe (N = 43)*		
		*Positive*		*Positive*		*Positive*		*Positive*			*Positive*		*Positive*		*Positive*		
**Variable**	**Cut-off**	*N*	*(%)*	*N*	*(%)*	*N*	*(%)*	*N*	*(%)*	*P trend*^a^	*N*	*(%)*	*N*	*(%)*	*N*	*(%)*	*P trend*^a^
Age (years)	≥55.5	252	(53)	79	(53)	49	(68)	26	(90)	<0.001	40	(48)	79	(64)	35	(81)	<0.001
*Hand/wrist measure*																	
Wrist ratio	≥0.708	195	(41)	83	(56)	44	(61)	21	(72)	<0.001	46	(55)	74	(60)	28	(65)	<0.001
Hand ratio	≤2.23	180	(38)	82	(55)	40	(56)	20	(69)	<0.001	48	(57)	67	(54)	27	(63)	<0.001
Shape index	≥44.8	180	(38)	82	(55)	40	(56)	20	(69)	<0.001	48	(57)	67	(54)	27	(63)	<0.001
Digit index	≥43.3	187	(39)	84	(56)	33	(46)	14	(48)	0.024	49	(58)	63	(51)	19	(44)	0.018
Wrist-palm ratio	≥0.385	195	(41)	78	(52)	46	(64)	27	(93)	<0.001	44	(52)	70	(57)	37	(86)	<0.001
Hand length/height	≤1.09	212	(45)	82	(55)	34	(47)	14	(48)	0.263	48	(57)	65	(53)	17	(40)	0.347
*Body measure*																	
Body mass index	≥24.8	194	(41)	85	(57)	46	(64)	24	(83)	<0.001	42	(50)	77	(63)	36	(84)	<0.001
A Body shape index	≥0.79	210	(44)	78	(52)	39	(54)	20	(69)	0.002	39	(46)	69	(56)	29	(67)	<0.001
Waist-hip ratio	≥0.85	189	(40)	83	(56)	45	(62)	21	(72)	<0.001	48	(57)	70	(57)	31	(72)	<0.001
Waist-stature ratio	≥0.54	178	(38)	85	(57)	45	(62)	23	(79)	<0.001	42	(50)	75	(61)	36	(84)	<0.001
Waist-hip-height ratio	≥0.53	196	(41)	85	(57)	51	(71)	25	(86)	<0.001	51	(61)	74	(60)	36	(84)	<0.001
	**MALES (N = 393)**																
				**Cases: clinical score**							**Cases: electrophysiological score**						
		**Controls** *(N = 273)*		*Mild (N = 58)*		*Moderate (N = 38)*		*Severe (N = 24)*			*Mild (N = 24)*		*Moderate (N = 64)*		*Severe (N = 32)*		
		*Positive*		*Positive*		*Positive*		*Positive*			*Positive*		*Positive*		*Positive*		
**Variable**	**Cut-off**	*N*	*(%)*	*N*	*(%)*	*N*	*(%)*	*N*	*(%)*	*P trend*^a^	*N*	*(%)*	*N*	*(%)*	*N*	*(%)*	*P trend*^a^
Age (years)	≥55.5	97	(36)	28	(48)	20	(53)	21	(87)	<0.001	8	(33)	34	(53)	27	(84)	<0.001
*Hand/wrist measure*																	
Wrist ratio	≥0.696	142	(52)	34	(59)	31	(82)	19	(79)	<0.001	15	(62)	47	(73)	22	(69)	0.001
Hand ratio	≤2.17	78	(29)	35	(60)	26	(68)	22	(92)	<0.001	13	(54)	45	(70)	25	(78)	<0.001
Shape index	≥46.1	78	(29)	35	(60)	26	(68)	22	(92)	<0.001	13	(54)	45	(70)	25	(78)	<0.001
Digit index	≥43.1	106	(39)	30	(52)	24	(63)	12	(50)	0.007	17	(71)	34	(53)	15	(47)	0.033
Wrist-palm ratio	≥0.397	68	(25)	28	(48)	30	(79)	23	(95)	<0.001	13	(54)	42	(66)	26	(81)	<0.001
Hand length/height	≤1.09	113	(41)	34	(59)	23	(61)	12	(50)	0.021	15	(62)	38	(59)	16	(50)	0.017
*Body measure*																	
Body mass index	≥26.9	98	(36)	31	(53)	25	(66)	20	(83)	<0.001	13	(54)	40	(62)	23	(72)	<0.001
A Body shape index	≥0.827	120	(44)	30	(52)	20	(53)	18	(75)	0.004	12	(50)	35	(55)	21	(66)	0.009
Waist-hip ratio	≥0.933	104	(38)	39	(67)	24	(63)	18	(75)	<0.001	17	(71)	44	(69)	29	(62)	<0.001
Waist-stature ratio	≥0.569	94	(34)	28	(48)	23	(61)	22	(92)	<0.001	12	(50)	37	(58)	24	(75)	<0.001
Waist-hip-height ratio	≥0.540	117	(43)	39	(67)	26	(68)	22	(92)	<0.001	16	(67)	45	(70)	26	(81)	<0.001

Classification of stages: mild, stage I-II; moderate, III; severe, stage IV-V.

^**a**^Score test for linear trend of the log odds across ordered groups.

Table 3 presented the measures of diagnostic accuracy of established cut-offs for the selected anthropometric indexes stratified by gender and clinical/electrophysiological severity scales. In particular, it should be noted that sensibility and specificity tended to increase with severity scale in both genders. Among females, the wrist-palm ratio showed a small but important change in the probability of disease in the severe stage with an estimated LR+ of 2.26 in the clinical score and 2.09 in the electrophysiological one; the LR- was 0.12 and 0.24, respectively. Among males, the LR+ for wrist-palm ratio was 3.85 in the severe stage of the clinical severity scale and 3.26 in the severe stage of the electrophysiological scale; the LR- was 0.06 and 0.25, respectively.

**Table 3:** Accuracy of established cut-offs by gender and clinical/electrophysiological score.

	**FEMALES (N = 724)**																							
	**Clinical score**												**Electrophysiological score**											
	**Mild**				**Moderate**				**Severe**				**Mild**				**Moderate**				**Severe**			
**Variable**	*SN*	*SP*	*LR+*	*LR-*	*SN*	*SP*	*LR+*	*LR-*	*SN*	*SP*	*LR+*	*LR-*	*SN*	*SP*	*LR+*	*LR-*	*SN*	*SP*	*LR+*	*LR-*	*SN*	*SP*	*LR+*	*LR-*
Age (years)	53	47	1.00	1.00	68	47	1.28	0.68	90	47	1.69	0.22	48	47	0.90	1.12	64	47	1.21	0.76	81	47	1.53	0.40
*Hand/wrist measure*																								
Wrist ratio	56	59	1.35	0.75	61	59	1.49	0.66	72	59	1.76	0.47	55	59	1.33	0.77	60	59	1.46	0.68	65	59	1.58	0.59
Hand ratio	55	62	1.45	0.72	56	62	1.46	0.72	69	62	1.82	0.50	57	62	1.50	0.69	54	62	1.43	0.73	63	62	1.65	0.60
Shape index	55	62	1.45	0.72	56	62	1.46	0.72	69	62	1.82	0.50	57	62	1.50	0.69	54	62	1.43	0.73	63	62	1.65	0.60
Digit index	56	61	1.43	0.72	46	61	1.16	0.89	48	61	1.22	0.85	58	61	1.48	0.69	51	61	1.30	0.81	44	61	1.12	0.92
Wrist-palm ratio	52	59	1.27	0.81	64	59	1.55	0.61	93	59	2.26	0.12	52	59	1.27	0.81	57	59	1.38	0.73	86	59	2.09	0.24
Hand length/height	55	55	1.23	0.81	47	55	1.06	0.95	48	55	1.08	0.94	57	55	1.28	0.78	53	55	1.18	0.85	40	55	0.88	1.09
*Body measure*																								
Body mass index	57	59	1.39	0.73	64	59	1.56	0.61	83	59	2.02	0.29	50	59	1.22	0.85	63	59	1.53	0.63	84	59	2.05	0.28
A Body shape index	52	56	1.18	0.86	54	56	1.22	0.82	69	56	1.56	0.56	46	56	1.05	0.96	56	56	1.27	0.79	67	56	1.52	0.58
Waist-hip ratio	56	60	1.40	0.74	63	60	1.57	0.62	72	60	1.82	0.46	57	60	1.43	0.71	57	60	1.43	0.72	72	60	1.81	0.46
Waist-stature ratio	57	62	1.52	0.69	63	62	1.66	0.60	79	62	2.11	0.33	50	62	1.33	0.80	61	62	1.62	0.62	84	62	2.23	0.26
Waist-hip-height ratio	57	59	1.38	0.73	71	59	1.71	0.50	86	59	2.08	0.24	61	59	1.47	0.67	60	59	1.45	0.68	84	59	2.02	0.28
	**MALES (N = 393)**																							
	**Clinical score**												**Electrophysiological score**											
	**Mild**				**Moderate**				**Severe**				**Mild**				**Moderate**				**Severe**			
**Variable**	*SN*	*SP*	*LR+*	*LR-*	*SN*	*SP*	*LR+*	*LR-*	*SN*	*SP*	*LR+*	*LR-*	*SN*	*SP*	*LR+*	*LR-*	*SN*	*SP*	*LR+*	*LR-*	*SN*	*SP*	*LR+*	*LR-*
Age (years)	48	64	1.36	0.80	53	64	1.48	0.73	88	64	2.46	0.19	33	64	0.94	1.03	53	64	1.50	0.73	84	64	2.37	0.24
*Hand/wrist measure*																								
Wrist ratio	59	48	1.13	0.86	82	48	1.57	0.38	79	48	1.52	0.43	63	48	1.20	0.78	73	48	1.41	0.55	69	48	1.32	0.65
Hand ratio	60	71	2.11	0.56	68	71	2.39	0.44	92	71	3.21	0.12	54	71	1.90	0.64	70	71	2.46	0.42	78	71	2.73	0.31
Shape index	60	71	2.11	0.56	68	71	2.39	0.44	92	71	3.21	0.12	54	71	1.90	0.64	70	71	2.46	0.42	78	71	2.73	0.31
Digit index	52	61	1.33	0.79	63	61	1.63	0.60	50	61	1.29	0.82	71	61	1.82	0.48	53	61	1.37	0.77	47	61	1.21	0.87
Wrist-palm ratio	48	75	1.94	0.69	79	75	3.17	0.28	96	75	3.85	0.06	54	75	2.17	0.61	66	75	2.63	0.46	81	75	3.26	0.25
Hand length/height	59	59	1.42	0.71	61	59	1.46	0.67	50	59	1.21	0.85	63	59	1.51	0.64	59	59	1.43	0.69	50	59	1.21	0.85
*Body measure*																								
Body mass index	53	64	1.49	0.73	66	64	1.83	0.53	83	64	2.32	0.26	54	64	1.51	0.71	63	64	1.74	0.58	72	64	2.00	0.44
A Body shape index	52	56	1.18	0.86	53	56	1.20	0.85	75	56	1.71	0.45	50	56	1.14	0.89	55	56	1.24	0.81	66	56	1.49	0.61
Waist-hip ratio	67	62	1.77	0.53	63	62	1.66	0.60	75	62	1.97	0.40	71	62	1.86	0.47	69	62	1.80	0.50	63	62	1.64	0.61
Waist-stature ratio	48	66	1.40	0.79	61	66	1.76	0.60	92	66	2.66	0.13	50	66	1.45	0.76	58	66	1.68	0.64	75	66	2.18	0.38
Waist-hip-height ratio	67	57	1.57	0.57	68	57	1.60	0.55	92	57	2.14	0.15	67	57	1.56	0.58	70	57	1.64	0.52	81	57	1.90	0.33

Abbreviations: SN, sensitivity (%); SP, specificity (%); LR+, positive likelihood ratio; LR-, negative likelihood ratio.

Classification of stages: mild, stage I-II; moderate, III; severe, stage IV-V.

The AUC and their 95% CI were reported in Table 4. The accuracy tended to increase with CTS severity for females and males. In the severe stage, most of the indexes presented moderate accuracy in both genders, although AUC were higher among males for almost all studied variables than in females. Among subjects with severe CTS, the wrist-palm ratio presented the highest AUC in the clinical and electrophysiological severity scales in females (AUC 0.83 and 0.76, respectively) and males (AUC 0.91 and 0.82, respectively). The diagnostic performance of the digit index and the hand length-height ratio did not improve across severity scales; these variables also showed the lowest AUC among subjects with severe disease. Among subjects with severe CTS, the waist-stature ratio showed the highest AUC for body measures in the clinical and electrophysiological severity scales both in females (AUC 0.78 and 0.77, respectively) and males (AUC 0.84 and 0.76, respectively).

**Table 4:** Area under the receiver operating characteristic curves by gender and clinical/electrophysiological score.

	**FEMALES (N = 724)**													
	**Clinical score**							**Electrophysiological score**						
	**Mild**		**Moderate**		**Severe**			**Mild**		**Moderate**		**Severe**		
**Variable**	*AUC*	*(95%CI)*	*AUC*	*(95%CI)*	*AUC*	*(95%CI)*	*P value*	*AUC*	*(95%CI)*	*AUC*	*(95%CI)*	*AUC*	*(95%CI)*	*P value*
Age (years)	0.49	(0.44–0.54)	0.56	(0.50–0.63)	0.75	(0.66–0.83)	<0.001	0.46	(0.40–0.52)	0.54	(0.49–0.59)	0.70	(0.63–0.78)	<0.001
*Hand/wrist measure*														
Wrist ratio	0.55	(0.49–0.60)	0.62	(0.56–0.68)	0.72	(0.63–0.80)	0.003	0.54	(0.47–0.60)	0.60	(0.54–0.65)	0.66	(0.58–0.74)	0.064
Hand ratio	0.60	(0.55–0.65)	0.61	(0.53–0.69)	0.73	(0.62–0.83)	0.108	0.61	(0.54–0.68)	0.60	(0.55–0.66)	0.68	(0.59–0.76)	0.355
Shape index	0.60	(0.55–0.65)	0.61	(0.53–0.69)	0.73	(0.62–0.83)	0.107	0.61	(0.54–0.68)	0.60	(0.55–0.66)	0.68	(0.59–0.76)	0.355
Digit index	0.60	(0.55–0.66)	0.51	(0.43–0.58)	0.51	(0.38–0.63)	0.075	0.62	(0.55–0.68)	0.55	(0.49–0.61)	0.50	(0.39–0.60)	0.137
Wrist-palm ratio	0.58	(0.53–0.63)	0.65	(0.59–0.72)	0.83	(0.77–0.89)	<0.001	0.58	(0.52–0.64)	0.62	(0.57–0.67)	0.76	(0.70–0.83)	<0.001
Hand length/height	0.55	(0.50–0.61)	0.52	(0.44–0.59)	0.53	(0.41–0.65)	0.722	0.58	(0.51–0.65)	0.53	(0.47–0.59)	0.50	(0.40–0.59)	0.316
*Body measure*														
Body mass index	0.61	(0.55–0.66)	0.65	(0.59–0.71)	0.73	(0.65–0.81)	0.048	0.57	(0.51–0.64)	0.64	(0.58–0.69)	0.74	(0.67–0.81)	0.002
A Body shape index	0.55	(0.50–0.60)	0.56	(0.49–0.63)	0.66	(0.56–0.77)	0.159	0.53	(0.46–0.59)	0.57	(0.51–0.62)	0.64	(0.56–0.73)	0.105
Waist-hip ratio	0.58	(0.53–0.63)	0.63	(0.56–0.69)	0.73	(0.65–0.82)	0.013	0.58	(0.51–0.64)	0.60	(0.54–0.65)	0.72	(0.65–0.79)	0.005
Waist-stature ratio	0.61	(0.55–0.66)	0.66	(0.60–0.73)	0.78	(0.70–0.86)	0.001	0.58	(0.51–0.64)	0.64	(0.59–0.70)	0.77	(0.71–0.83)	<0.001
Waist-hip-height ratio	0.57	(0.52–0.63)	0.65	(0.58–0.71)	0.78	(0.70–0.86)	<0.001	0.58	(0.51–0.64)	0.60	(0.55–0.66)	0.75	(0.68–0.82)	<0.001
	**MALES (N = 393)**													
	**Clinical score**							**Electrophysiological score**						
	**Mild**		**Moderate**		**Severe**			**Mild**		**Moderate**		**Severe**		
**Variable**	*AUC*	*(95%CI)*	*AUC*	*(95%CI)*	*AUC*	*(95%CI)*	*P value*	*AUC*	*(95%CI)*	*AUC*	*(95%CI)*	*AUC*	*(95%CI)*	*P value*
Age (years)	0.57	(0.49–0.65)	0.59	(0.49–0.68)	0.88	(0.81–0.94)	<0.001	0.47	(0.35–0.58)	0.61	(0.54–0.69)	0.81	(0.73–0.89)	<0.001
*Hand/wrist measure*														
Wrist ratio	0.54	(0.46–0.61)	0.69	(0.59–0.78)	0.64	(0.54–0.74)	0.040	0.54	(0.43–0.66)	0.63	(0.56–0.71)	0.60	(0.50–0.71)	0.415
Hand ratio	0.73	(0.66–0.79)	0.73	(0.64–0.83)	0.86	(0.78–0.94)	0.031	0.70	(0.60–0.81)	0.76	(0.69–0.83)	0.78	(0.69–0.88)	0.519
Shape index	0.73	(0.66–0.79)	0.73	(0.64–0.83)	0.86	(0.78–0.94)	0.031	0.70	(0.60–0.81)	0.76	(0.69–0.83)	0.78	(0.69–0.88)	0.521
Digit index	0.59	(0.48–0.64)	0.60	(0.51–0.70)	0.52	(0.38–0.66)	0.580	0.66	(0.54–0.77)	0.56	(0.48–0.63)	0.51	(0.39–0.63)	0.193
Wrist-palm ratio	0.66	(0.58–0.73)	0.80	(0.72–0.88)	0.91	(0.85–0.97)	<0.001	0.65	(0.52–0.77)	0.76	(0.69–0.83)	0.82	(0.73–0.90)	0.076
Hand length/height	0.58	(0.50–0.65)	0.59	(0.49–0.69)	0.56	(0.44–0.67)	0.934	0.59	(0.47–0.71)	0.59	(0.51–0.66)	0.55	(0.45–0.65)	0.793
*Body measure*														
Body mass index	0.61	(0.53–0.69)	0.65	(0.56–0.74)	0.78	(0.71–0.86)	0.005	0.60	(0.49–0.71)	0.66	(0.58–0.73)	0.70	(0.61–0.79)	0.380
A Body shape index	0.55	(0.47–0.62)	0.52	(0.42–0.62)	0.70	(0.58–0.81)	0.042	0.52	(0.40–0.63)	0.54	(0.46–0.62)	0.66	(0.56–0.75)	0.116
Waist-hip ratio	0.64	(0.57–0.72)	0.68	(0.59–0.77)	0.72	(0.62–0.82)	0.486	0.65	(0.52–0.78)	0.68	(0.61–0.74)	0.68	(0.59–0.77)	0.916
Waist-stature ratio	0.63	(0.55–0.70)	0.62	(0.53–0.71)	0.84	(0.76–0.93)	<0.001	0.59	(0.47–0.70)	0.65	(0.58–0.73)	0.76	(0.67–0.85)	0.055
Waist-hip-height ratio	0.67	(0.59–0.74)	0.64	(0.55–0.73)	0.82	(0.73–0.90)	0.009	0.64	(0.52–0.76)	0.67	(0.60–0.74)	0.75	(0.67–0.84)	0.222

Abbreviations: AUC, area under the curve; 95%CI, 95% confidence intervals.

Classification of stages: mild, stage I-II; moderate, III; severe, stage IV-V.

**Fig 1:**
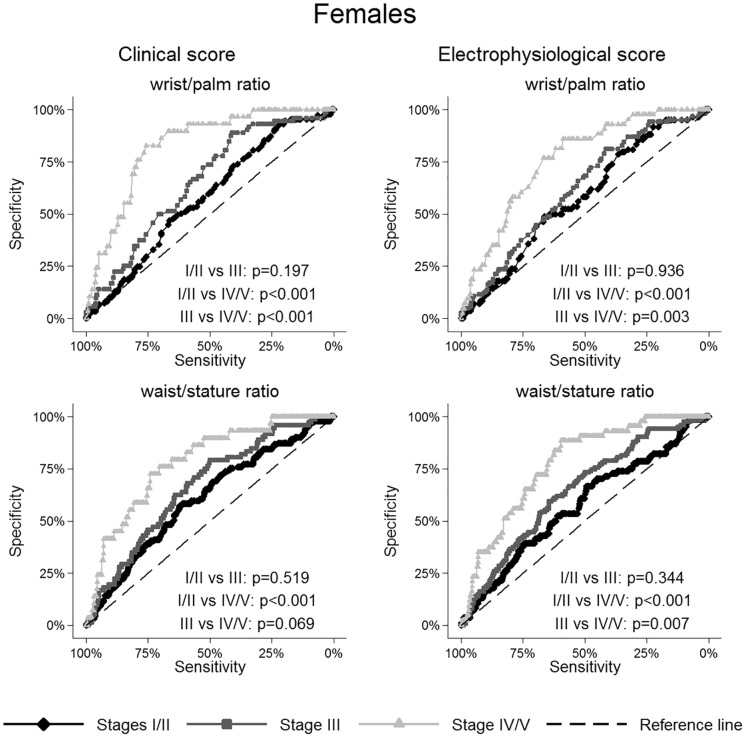
Receiver operating characteristic curves for selected anthropometric variables in females according to clinical and electrophysiological severity scales.

**Fig 2:**
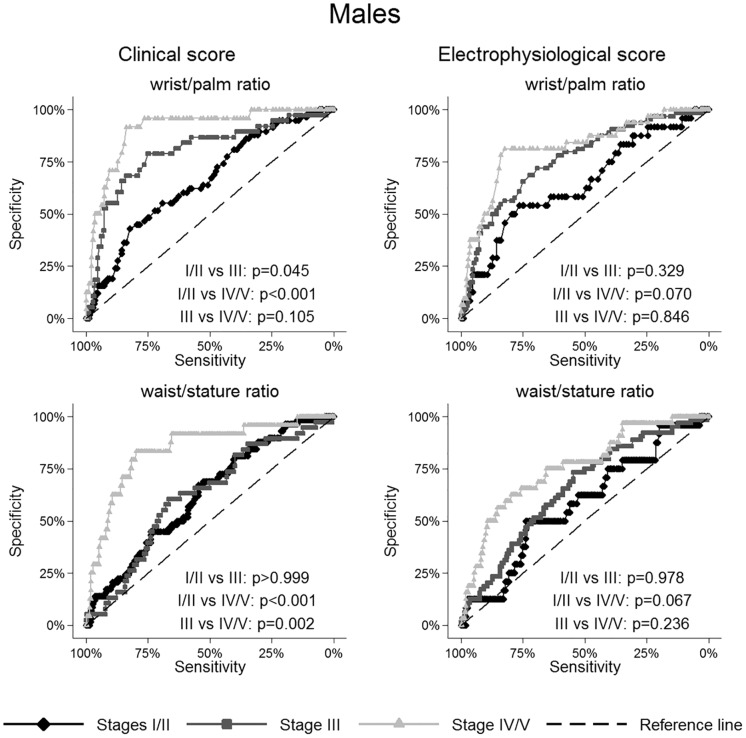
Receiver operating characteristic curves for selected anthropometric variables in males according to clinical and electrophysiological severity scales.

The ROC curves of wrist-palm ratio and waist-stature ratio were reported in Figs 1 and 2 for females and males, respectively.

## Discussion

Our study evaluates the diagnostic properties of hand/wrist and body measures according to validated clinical and electrophysiological CTS severity scales. We demonstrated that the accuracy of body and hand measures varied with the clinical and electrophysiological severity of CTS.

For the selected anthropometric measurements, the accuracy tended to increase with CTS severity for clinical and electrophysiological scales. Most of the indexes evaluated in the present study reported a moderate accuracy for discriminating patients with severe CTS from controls. In particular, their discriminatory power tended to be higher in males than in females. Moreover, in the cases with severe CTS, the discriminatory power of these anthropometric measurements seems to be higher when considering the clinical severity scale [29] with respect to the electrophysiological one [30].

Among hand/wrist anthropometric measures, the wrist-palm ratio reported moderate/high accuracy in the severe stage of CTS for both clinical and electrophysiological severity scale among males and females. In the severe stage of CTS for both severity scales, the waist-hip-height ratio and waist-stature ratio were the indexes with the highest capacity to discriminate patients with and without CTS as compared to other body measurements.

In the previous study we found that body/hand measures and their ratios showed limited accuracy for discriminating CTS cases (irrespective of severity) from controls, especially in females [25]. On the contrary, in the present study these anthropometric measurements show moderate/high accuracy in the case of the identification of patients with a severe stage of CTS. Thus, the studied anthropometric characteristics seem to have a good potential as screening test to help to identify subjects with severe CTS.

For many years BMI was used as an indicator of dangerous obesity. In actual fact, BMI does not distinguish between muscle and fat accumulation and between fat localization. These differences are not pleonastic as there is evidence that whereas higher fat mass is associated with greater risk of premature death, higher muscle mass reduces the risk; moreover, central or abdominal fat deposition is thought to be particularly perilous [31]. Over time, it is recognized that the risk related to the dangerous obesity was also affected by different body shapes, and new obesity indicators was considered, including waist-hip-height ratio and waist-stature ratio [31]. These indicators, unlike the BMI, give information about the distribution of body fat and are correlated with abdominal obesity.

With respect to the CTS it is widely known the relationship between BMI and electrophysiological conduction parameters of the median nerve [8,11]. In present paper we showed that in the severe stage of CTS, new obesity indicators (waist-hip-height ratio and waist-stature ratio) had higher capacity to discriminate patients with severe CTS than BMI. This finding certainly enhances the interest towards these new obesity indicators.

The associations between selected anthropometric and obesity indexes and CTS severity have been recently reported [35]. The authors found that all the studied hand and wrist indexes were associated with both clinical and electrophysiological severity. Whereas, among the adiposity indexes, the waist/stature ratio showed the strongest association with CTS severity [35].

Future studies with a proper longitudinal study design could be conducted to determine if weight loss and the reduction of the abdominal obesity could influence a recovery in median nerve conduction velocity in CTS subjects with obesity or reduce the severity of CTS. To date only one article has been addressed to this aim, but the patients in severe electrophysiological CTS stage were excluded from the study, making the result of the paper not fully conclusive [36].

A variety of clinical diagnostic tests has been proposed for CTS [37], however the clinical value of each single test was found to be limited [38,39]. The diagnostic value of a clinical prediction rule (CPR) has been demonstrated for many disorders [40,41]. The development of a CPR for the diagnosis of CTS would be extremely valuable [42], since it could increase physicians’ diagnostic accuracy.

Considering that the sensitivity of provocative clinical tests was reported to be lower in advanced stages of clinical and electrophysiological CTS severity [43], the results of this study may be useful to the development of a CPR for CTS, in the different severity stages of the disease.

The main strength and limits of the study have extensively been reported elsewhere [26,27]. In brief, selected indicators were calculated and their measure was based on standardized and reproducible methods. A restrictive case definition was used, since it comprised the coexistence of symptoms, clinical signs and electrophysiological abnormalities. Subjects with CTS symptoms and normal electrodiagnostic tests, and subjects with asymptomatic delay of distal conduction velocity of the median nerve were excluded from the study.

We enrolled the controls among the patients admitted to the same EMG labs as cases because of upper limb complaints other than CTS. We cannot exclude selection bias of controls, even though a lot of them had no disorders of the peripheral nervous system and the others suffered from diseases in which association with hand conformation and body characteristics are not known.

The analysis was stratified by gender, since the anthropometric measures are different among males and females. It should be underlined that the suggestive findings emerging from the present work should be further investigated in appropriately sized studies (especially for males).

In conclusion, the studied anthropometric measures–especially wrist-palm ratio, waist-hip-height ratio and waist-stature ratio–could be useful to support the diagnostic hypothesis of severe CTS, that has to be confirmed by nerve conduction study. They could be considered a possible predictor of CTS worsening–a condition that deserves special attention in the management of this neurological disorder.

## Supporting Information

S1 Table Database used for the statistical analysis. (XLS)
